# Proteomic Analysis of Porcine Pre-ovulatory Follicle Differentiation Into Corpus Luteum

**DOI:** 10.3389/fendo.2019.00774

**Published:** 2019-11-15

**Authors:** Pawel Likszo, Dariusz J. Skarzynski, Beenu Moza Jalali

**Affiliations:** Institute of Animal Reproduction and Food Research, Polish Academy of Sciences, Olsztyn, Poland

**Keywords:** preovulatory follicles, luteinization, corpus luteum, porcine, proteomics

## Abstract

The luteinization of the follicular cells, following a LH surge, causes extensive molecular and structural changes in preovulatory follicles (POF) that lead to ovulation and ultimate formation of the corpus luteum (CL). The objective of this study was to identify proteins expressed in porcine POF before the LH surge and a new CL formed, 2–3 days after ovulation, and evaluate proteome changes associated with formation of the CL from a follicle. We used 2D-gel electrophoresis-based proteomics and tandem mass spectrometry followed by a functional analysis using Ingenuity Pathway analysis (IPA) to evaluate functional pathways associated with the luteinization process. Protein lysates were prepared from isolated POFs and from the newly formed CL. A total of 422 protein spots were identified in both structures. A total of 15 and 48 proteins or their proteoforms were detected only in the POFs and CL, respectively. An IPA analysis of a POF proteome showed that most of the follicular proteins were involved in cellular infiltration, endoplasmic stress responses, and the protein ubiquitination pathway. Most of the early luteal proteins were associated with steroid metabolism, cell death and survival, free radical scavenging, and the protein ubiquitination pathway. A comparison of a follicular proteome with that of an early luteal proteome revealed that 167 identified proteins or their proteoforms were differentially regulated between POFs and the newly formed CL (*p* < 0.05 and a fold change of >1.8). Proteins that were significantly more abundant in follicles included cAMP-dependent protein kinase, histone binding protein RBBP4, reticulocalbin, vimentin, and calumenin; more abundant luteal proteins included albumin, farnesyl diphosphate synthase, serine protease inhibitors, elongation factor-1, glutaredoxin, and selenium-binding protein. Proteins that were significantly altered with luteal formation were found to be associated with cholesterol biosynthesis, cell death and survival, and acute phase response. Moreover, upstream regulators of differentially abundant proteins in CL were identified that included insulin growth factor-1, sterol regulatory element-binding transcription factor-1, and nuclear factor erythroid-derived 2. We have identified novel proteins that advance our understanding of (1) processes associated with differentiation of POFs into the CL, (2) possible mechanisms of luteal cell survival, and (3) pathways regulating steroidogenesis in the newly formed CL.

## Introduction

Reproductive efficiency in mammals is dependent on a well-coordinated ovarian cycle that includes follicular development, ovulation, corpus luteum formation, function, and regression in the absence of a pregnancy. The ovulation after the luteinizing hormone (LH) surge leads to the release of the oocyte and differentiation of the ruptured follicle (granulosa and theca cells) into luteal cells of the corpus luteum (CL), a progesterone (P4)-producing transient endocrine gland essential for the establishment and maintenance of early pregnancy. Luteinization primarily involves structural remodeling and changes in the expression of proteins that would initiate differentiation of estrogen-producing granulosa cells to the P4-producing luteal cells. The preovulatory follicles (POF)s under the influence of follicle stimulating hormone (FSH), LH, and growth factors undergo cell proliferation, support oocyte development, and synthesize estrogen (1–6). However, after the LH surge, granulosa cells cease to proliferate and express a different set of genes/proteins to support the luteal phenotype and P4 synthesis (7). The differentiation of the ruptured follicle to the CL is accompanied by changes in the extracellular matrix that allows cell migration. The LH surge induces expression of several MMPs and TIMPs facilitating follicle rupture during ovulation in many species, including pigs (8, 9). The LH also results in the increased expression of growth and angiogenic factors, such as the vascular endothelial growth factor (VEGF), angiopoietin, and their receptor to induce vascularization of the newly formed CL after luteinization (10, 11).

The targeted studies carried out in various species, especially in rodents, using gene knock-out models has elucidated the importance of many genes in the CL formation. Luteinization has been shown to be associated with a change in the expression of genes involved in the cell cycle, such as cyclin-dependent kinases, ovulation, such as cyclooxygenase-2, CATT/enhancer binding protein β (C/EBP), and the P4 receptor, and in cAMP/PKA signaling (12–14). The transcriptomics data related to the luteinization of follicular cells in mouse, rat, bovine, and porcine species has also contributed immensely to our understanding of processes associated with the luteinization of follicular cells (15–19). Despite many similarities between the follicular development, steroidogenesis, and ovulation across the species, there are species specific differences in these processes (20). In pigs, although there have been many reports detailing molecular changes associated with POF development and growth (21–24) and corpus luteum function and regression [reviewed in Ziecik et al. (25)], the studies regarding the luteinization of porcine follicles and CL formation are scarce (26, 27). There is, however, one study detailing the global changes in gene expression of porcine POFs after their luteinization (19); a proteomic analysis of changes associated with formation of the CL from POFs has not been reported yet. The luteinization of granulosa cells is known to start before ovulation (19); however, there are not many reports of regulatory mechanisms associated with ovulation and CL formation. As the formation of CL is essential to the success of pregnancy establishment, a detailed evaluation of molecular changes associated with CL formation was carried out using two-dimensional gel electrophoresis (2D-PAGE) followed by a functional analysis using Ingenuity Pathway analysis to identify processes that differ between these two structures. Owing to the use of porcine models in studies related to ovarian steroidogenesis, biomedical research (28), and a high degree of similarity in protein coding sequences between humans and pig, such a proteomic database might potentially allow for addressing causes of ovulation-related disorders in women. We identified key molecules associated with CL formation, survival, and steroidogenesis, and several novel proteins associated with steroidogenesis were also identified in this study.

## Materials and Methods

### Animals

All procedures involving the use of animals were approved by the Animal Ethics Committee, University of Warmia and Mazury in Olsztyn, Poland, and were conducted in accordance with the national guidelines for agricultural animal care. Porcine ovaries were collected after the animals were slaughtered at a local abattoir and transported to the laboratory in phosphate-buffered saline (PBS) at room temperature. Ovaries were classified using morphological criteria described previously (29). Moreover, the follicular fluid concentrations of hormones estradiol-17 b (E2) and P4 were determined to classify the follicles as POFs before the LH surge. Four to five individual pre-ovulatory follicles more than 6 mm in diameter and that were from one pig were isolated by mechanical dissection. The follicles were punctured and the follicular fluid (FF) was aspirated using an 18-gauge needle fixed to a 5-ml syringe. The oocytes were removed and FF samples were centrifuged at 2,000 g for 15 min at 4°C. Supernatants were analyzed for E2 and P4 concentration using a radioimmunoassay kit (Cisbio International) according to manufacturer's instructions, and the E2:P4 ratios were calculated for each sample. The pre-ovulatory follicular walls from the same animal with E2 to P4 ratio >1.0 were pooled together and snap frozen in liquid nitrogen and stored at −80°C until protein lysate preparation. The CL were collected after 2–3 days of ovulation, characterized morphologically (29), snap frozen and stored at −80°C till further analysis. The POFs and CL used in this study were collected from four individual animals (*n* = 4).

### Sample Preparation

Total protein was isolated from 4 to 5 pre-ovulatory follicles collected from same animal and pooled together (*n* = 4), as were the newly formed CL (*n* = 4). The samples were homogenized using a ceramic mortar and pestle and then precooled with liquid nitrogen for at least 1 min. Homogenized frozen tissue was directly transferred into a lysis buffer (30 mM Tris-HCL, 7M urea, 2M thiourea, 4% w/v CHAPS and protease inhibitor). Lysates were sonicated for 4 min in a Sonics Vibra-Cell VCX 120 and centrifuged in a Beckman Ultracentrifuge J2-HS for 30 min at 2,000 g and at 4°C. Protein concentrations were determined using the Bradford method.

### Two-Dimensional Gel Electrophoresis (2-DE)

Protein lysates (600 μg) from preovulatory follicles and CL were suspended in rehydration buffer (7M urea, 2M thiourea, 2% w/v CHAPS, 10 mM DTT, 1% v/v IPG buffer pH 4–7 and 0.002% bromophenol blue) in a final volume of 340 μl. The protein samples were loaded on 18 cm Immobiline DryStrips, pH 4–7 (GE Healthcare, Uppsala, Sweden), and rehydrated for 10 h (passive rehydration). The rehydrated strips were focused at 50 μA per strip in an Ettan IPGphor IEF System I (GE Healthcare, Uppsala, Sweden) with the following voltage program: 500 V for 8 h, 1,000 V for 1 h, 8,000 V for 3 h, and 8,000 V for 2.5 h. Prior to gel electrophoresis, focused proteins in the IPG strips were equilibrated in two incubation steps, each lasting 15 min, at room temperature with slow shaking. In the first step, each strip was equilibrated in 10 mL of equilibration buffer (50 mM Tris-HCl pH 6.8, 6M urea, 30% v/v glycerin, 2% w/v SDS and trace of bromophenol blue) supplemented with 1% w/v DTT. The second equilibration step involved alkylation in the same equilibration buffer that contained 2.5% w/v iodoacetamide instead of DTT. For the second dimension analysis, strips were applied onto 12.5% polyacrylamide gels and sealed with 0.5% agarose. The second electrophoresis was run (Bio-Rad) at 40 mA for 30 min, 60 mA for 1.5 h, and 80 mA for 2 h at 4°C. After electrophoresis, gels were fixed in methanol:acetic-acid:water (40:10:50) for 1 h followed by staining using a Coomassive Brilliant Blue G250 (Sigma Aldrich, Saint Louis, USA). The gels were destained and scanned with an ImageScanner II (GE Healthcare).

### Image and Data Analysis

The gel images were analyzed using the ImageMaster 2-D Platinum software version 7 from GE Healthcare. For comparison of protein spots between pre-ovulatory follicles and the CL, more than 15 spots in all gels were landmarked and normalized. Only protein spots with reproducible change of a minimum of 1.8-fold increase or decrease in their relative abundance and *p* < 0.05 by one way Anova in four biological replicates were considered significantly altered in the CL as compared to the pre-ovulatory follicle. Each group was performed in triplicate.

### Excision of 2D-gel Spots, Tryptic Digestion and MALDI-TOF/TOF Analysis

To define the overall proteome of pre-ovulatory follicles and newly formed CL, clearly visible spots were manually picked from gels and washed for 30 min each in distilled water followed by 50 mM ammonium bicarbonate. Protein spots were digested overnight at 37°C using an in-gel tryptic digestion kit (Thermoscientific, MA) and following manufacturer's instructions. After digestion, the peptides were suspended in 100 μl of 0.1% trifluoroacetic acid (TFA) and desalted using C-18 zip tips (Sigma-Aldrich). The peptides were eluted from the zip tips with 2 μl of 50% acetonitrile in 0.1% TFA and mixed 1: 1 with matrix solution (5 mg/ml solution of α-cyano-4-hydroxycinnamic acid in 50% acetonitrile/0.1% TFA). 1 μl of peptide-matrix mixture was spotted on the MALDI target plate and left to dry at room temperature. The digested peptides were analyzed with a MALDI-MS/MS mass spectrometer, Autoflex-TOF/TOF (Bruker Daltonics, Bremen, Germany) in positive ion reflector mode with an accelerating potential at 20 kV with eight shots per second was also used. The mass spectra were internally calibrated using monoisotpoic [M+H]+ ion peptide calibration standards (Bruker Daltonics) consisting of Angiotensin II (1046.54), Angiotensin I (1296.68), Substance P (1347.73), Bombesin (1619.82), ACTH clip 1 (2093.086), ACTH clip 18 (2465.19), and Somatostatin 28 (3147.471). Mass spectra were processed with the Flex Analysis and Biotool 2.2 software (Bruker Daltonics). Peptide mass finger printing (PMF) and fragment mass spectra (MS/MS) for each individual spot were combined, and an ion search was performed with the MASCOT 2.2 software (Matrix Science) integrated with Biotool 2.2. The Mass Spectrometry Protein Sequence Database (Version 09292005; 2,344,227 sequences) and the National Center for Biotechnology Information non-redundant Sus scrofa protein database were searched with the following MASCOT settings: one incomplete tryptic cleavage allowed; fragment ion mass tolerance of 0.8 Da; parent ion mass tolerance of 0.8 Da; alkylation of cysteine by carbamidomethylation as a fixed odification; oxidation of methionine as a variable modification. For the PMF and MS/MS ion search, statistically significant (*p* ≤ 0.05) matches by MASCOT were regarded as correct hits. We identified a total of 451 spots including the proteins that were differentially regulated between pre-ovulatory follicles and the CL. A complete proteomic work flow is depicted in Supplementary Figure 1.

### Ingenuity Pathway Analysis

IPA (Ingenuity, Mountain View, CA) was used to gain insights into the involvement of all the identified follicular, luteal, and differentially abundant proteins between these two structures in biological pathways and networks. IPA identifies networks of interacting proteins and connects identified proteins in the data set to molecular networks contained within the Ingenuity Knowledge Database. The identified proteins were analyzed using IPA Core analysis to assess functional pathways and upstream regulators that were over represented in the data set. Fisher's exact test was used to calculate a *P*-value for each network and a functional pathway to determine which pathways are significantly linked to input data that is mapped to genes/proteins in the whole Ingenuity Pathways Knowledgebase. We also used IPA Upstream Regulator analysis to identify upstream transcription regulators by Fisher's exact *t*-test. The right-tailed Fisher's exact test, using a threshold of *P* < 0.05 after application of the Benjamini-Hochberg method for multiple testing correction, and z-score (in case of proteins with significantly altered abundances) were used as two statistical measures for identifying significant biofunctions and upstream regulators. The *z*-score value indicated the state of activation (*z*-score ≥ 2.0) for the analyses. The Upstream Regulator module identified the upstream regulators that might explain the observed changes in protein abundances in our dataset and highlight the biological activities occurring during the luteinization process.

### Western Blot

Western-blot analysis was performed to validate the identity of select proteins belonging to categories unfolded protein response, oxidative stress response, cell proliferation and migration, sterol transport, and to quantitatively assess their abundance in select samples. Total protein lysate (25 μg) from a sample of POFs and the CL were resolved by 8% and 10% SDS-PAGE gel electrophoresis. Proteins were transferred onto polyvinylidene difluoride membranes (0.45 μm; Sigma-Aldrich, Saint Louis, USA) at 60 V for 90 min. Membranes were blocked with 5% non-fat milk in TBS-T (Tris buffered saline plus 0.05% Tween 20, pH 7.4) for 1.5 h at room temperature. Subsequently, membranes were washed thrice with TBS-T and incubated with following primary antibodies: rabbit anti-porcine ceruloplasmin, rabbit anti-porcine ERp57 (PDIA3), rabbit anti-porcine vitamin D binding protein (VDBP), mouse anti-porcine selenium binding protein 1 (SELENBP1), rabbit anti-porcine β-actin (Abcam, Cambridge, UK), rabbit anti-porcine aldose reductase (AKR1B1), and mouse anti-porcine heat shock protein (HSP) 60 (Sigma-Aldrich, Saint Louis, USA) diluted in TBS-T at 4°C overnight. The following day, the membranes were washed three times with TBS-T and incubated with anti-rabbit (Sigma-Aldrich, Saint Louis, USA) or anti-mouse (Abcam, Cambridge, UK) polyclonal secondary antibodies conjugated with alkaline phosphatase, diluted in TBS-T for 1.5 h at room temperature. The membranes were washed three times and protein bands were visualized by incubation in a solution of alkaline phosphate buffer (100 mM Tris-HCL, pH 9.5; 100 mM NaCl; 5 mM MgCl_2_) with an addition of Nitro Blue Tetrazolium (Sigma-Aldrich, Saint Louis, USA) and 5-bromo-4-chloro 3-indolyl phosphate (Sigma-Aldrich, Saint Louis, USA) in the dark. The membranes were washed in deionized water for 1 min to stop the reaction color. The intensity of the protein bands was quantified by measuring optical density using a ChemiDoc™ Touch Imaging System (Bio-Rad, Hercules, CAL, USA). The signal was analyzed using the Image Lab version 5.2 (Bio-Rad, Hercules, CAL, USA) and normalized to β-actin.

### Statistical Analysis

The statistical analysis of the Western Blot data was performed by using GraphPad PRISM v.7.0 software (GraphPad Software, Inc., San Diego, CA, USA). To test the changes in the expression of the selected proteins between POFs and the newly formed CL and validate the proteomic results, an unpaired Student's *t*-test followed by a Mann-Whitney post-test were applied with the significance set at *P* < 0.05.

## Results

### Hormone Assay in Follicular Fluid

The concentration of E2 and P4 in follicular fluid pooled from 5 follicles ranged from 161.41 ± 34.70 ng/mL, 208 ± 36 ng/ml, 251 ± 42 ng/ml, and 229 ± 24 ng/ml for E2 and 109.04 ± 3.28 ng/ml, 168.21 ± 2.9 ng/ml, 176.73 ± 17.23, and 202 ± 2.08 ng/ml for P4, respectively, in four different animals. Follicles were regarded as pre-ovulatory estrogenic when the E2/P4 ratio was more than 1.0 (19).

### Characterization of Pre-ovulatory Follicular and Newly Formed Corpus Luteum Proteome

**Figure 1:**
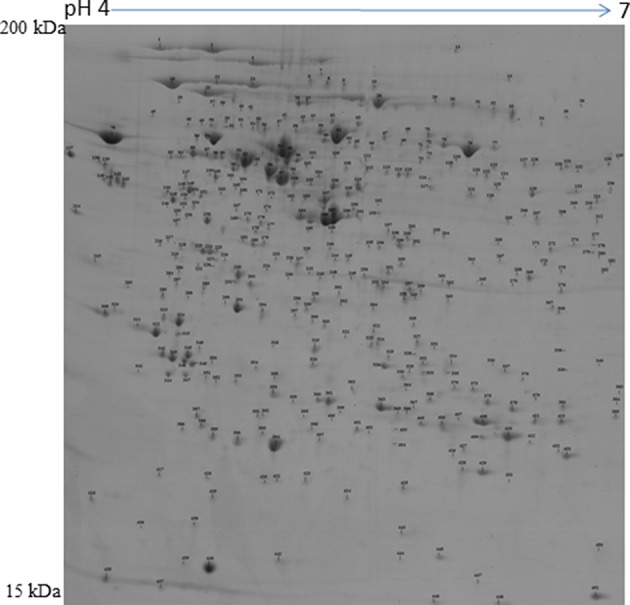
Representative protein map of the porcine preovulatory follicles. Proteins corresponding to spot numbers are listed in Supplementary Table 1.

A 2D-PAGE of the protein lysates from pooled follicles (>6 mm in size) from the same animal and the CL obtained from porcine ovaries after 2–3 days of ovulation was performed over a pH range of 4–7. Analysis of the 2-DE gels with ImageMasterTM 2D Platinum Software led to visualization of ~600 spots. The protein spots that were visualized in at least three gels were selected for identification by mass spectrometry. A total of 422 spots corresponding to proteins and their proteforms were identified in the POFs and CL using MALDI-MS/MS analysis (Figure 1 and Supplementary Tables 1, 2). The follicular proteome was predominantly represented by structural proteins: actin, myosin, vimentin, and tubulin; calcium binding proteins: calreticulin, calumenin, and reticulocalbin, and proteins involved in ubiquitin-proteasome system: 26S proteasome non-ATPase regulatory subunit 13 and ubiquitin carboxyl-terminal hydrolase isozyme L1. For functional analysis using Ingenuity Pathway Analysis (IPA), protein IDs were submitted to Biomart and converted to 378 human specific Ensembl gene IDs (http://useast.ensembl.org/biomart). The remaining proteins did not map to human Ensembl gene IDs because they were uncharacterized proteins or unique to *Sus scrofa* (e.g., apolipoprotein R). A functional analysis of all of the POF proteins using IPA revealed that top processes that proteins were associated with included protein ubiquitination (20 proteins), endoplasmic stress response (11 proteins) remodeling of cytoskeleton (7 proteins), and unfolded protein response (7 proteins) (Table 1). The luteal proteome was highly represented by protease inhibitors (serpins and cathepsins) and proteins involved in the transport and synthesis of lipids [steroidogenic acute regulatory protein (STAR), farnesyl diphosphate synthase (FDPS), and aldo-keto reductase family 1 member C4 (AKR1C4)]. The IPA identified proteins that were associated with pathways such as the protein ubiquitination pathway (23 proteins), steroid metabolism (16 proteins), NRF2-mediated oxidative stress response (14 proteins), and acute phase response signaling (14 proteins) (Table 1).

**Table 1:** Functional categories and their associated proteins that are highly represented in POFs and the corpus luteum.

**Functional categories**	**Associated proteins**
**POFs**	
Protein ubiquitination	HSP90AA1, HSP90AB1, GRP78, HSPA8, HSPB1, HSP60, GRP78, PSMA3, PSMA6, PSMB3, PSMD11, SUGT1, TRAP1, UBE2K, PSME2, UCHL1, UCHL3, USP14, PSMD5, PSMD13
Endoplasmic reticulum stress response	HSP90AA1, HSP90AS1, GRP78, GRP94, HSPD1, P4HP, SOD1, UCHL1, ERP44, GORASP2, VCP
Unfolded protein response	CALR, PDIA6, CALU, P4HP, HSPA5, HSPA8, VCP
Remodeling of cytoskeleton	ACTB, ACTG1, ARP5, ACT2, MAPRE1, TUBB, VIM
**NEWLY FORMED CORPUS LUTEUM**	
Protein ubiquitination	HSP90AA1, HSP90AB1, HSPA9, HSPB1, HSPD1,GRP78, HSPA8, HSPB1, HSP60, GRP78, PSMA3, PSMA6, PSMB3, PSMD11, PSME2, PSMD13, SUGT1, TRAP1, UBE2K, UCHL1, UCHL3, USP14, PSMD5
Steroid synthesis	AKR1C1/C2, AKR1C4, APOA1, APOE, CYB5A, FDPS, IDI1, ODC1, PHB, SET, StAR, G6PD, HMGCS1, HSD17B1, HSD3B1, HSD3B2
NRF2-mediated oxidative stress response	CLPP, FTH, FTL, SOD1, GST, MAPK3, VCP, ACTB, ACG1, USP4, CP, PRDX6, PRDX4, GPX3,
Acute phase response	FGG, FTL, HNRNPK, HP, HPX, RBP4, SERPINA3, AHSG, ALB, CRP, CP, APOA1, C4BPA, MAPK3

### Characterization of Proteins Associated With Differentiation of Follicles to CL

**Figure 2:**
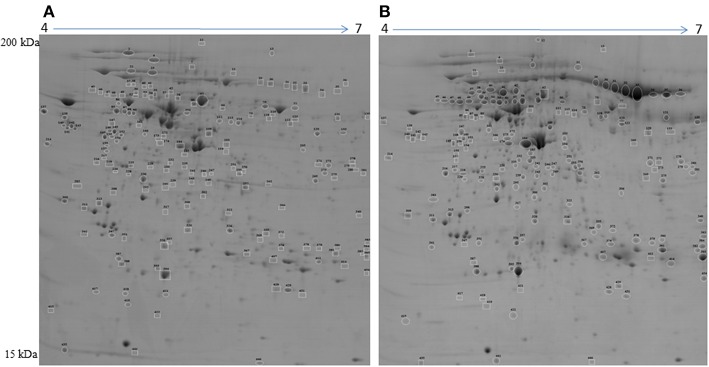
Representative 2D gel image illustrating comparison between **(A)** preovulatory follicles and **(B)** newly formed corpus luteum proteomes. Significantly altered spots (*P* < 0.05) with at least 1.8-fold changes in intensity were identified as upregulated (circles) or downregulated (squares) in POFs or the CL. Spot numbers correspond to the proteins that were identified by mass spectrometry analysis and are presented in Table 2. Images are representative of four gel analyses.

**Figure 3:**
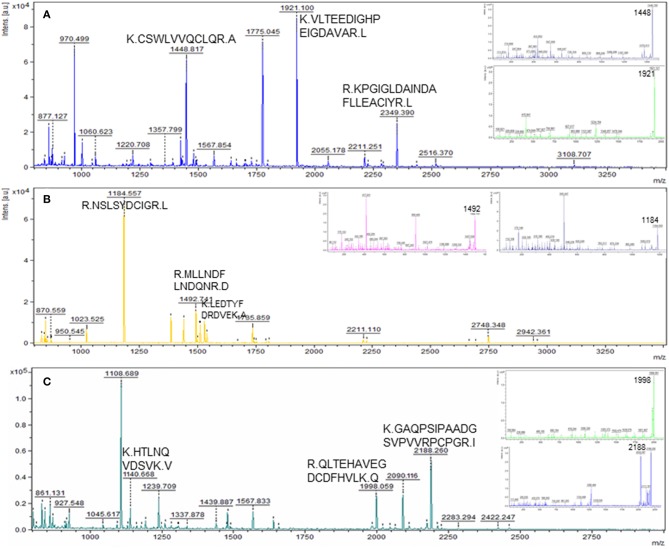
Representative MS and MS/MS (insets) spectra of some of the novel differentially abundant proteins in the CL as compared to POF. Matched peptides with a significant ion score of >30 are shown. **(A)** Peptides KCSWLVVQCLQRA, KVLTEEDIGHPEIGDAVARL, and RKPGIGLDAINDAFLLEACIYRL matched to protein FDPS; **(B)** peptides RNSLSYDCIGRL, RMLLNDFLNDQ NRD, and KLEDTYFDRDVEKA matched to protein HMGCS1; **(C)** peptides KHTLNQVDSV KV, RQLTEHAVEGDCDFHVLKQ, and KGAQPSIPAADGSVPVVRPCPGRI matched to protein AHSG.

**Figure 4:**
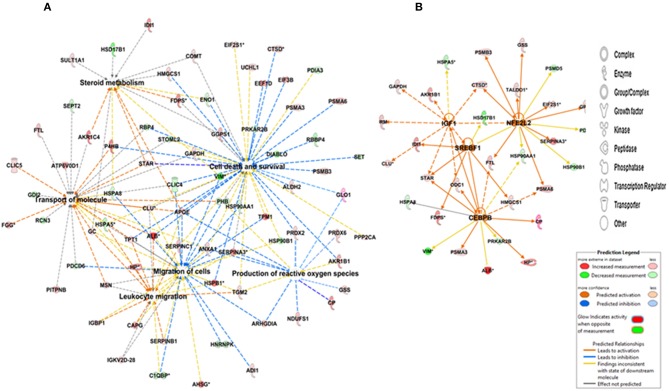
IPA depicting **(A)** networks integrating proteins involved in cell death and survival, cell migration, the scavenging of reactive oxygen species, and lipid metabolism and **(B)** upstream regulators of differentially abundant proteins in the CL. Red and green colors depict an increase or decrease, respectively, in abundance of the proteins in the CL. The color intensity of nodes indicates a fold change increase or decrease associated with a particular protein.

To detect changes associated with the transition of POFs to the CL after the LH surge, the protein profiles of POFs and the newly formed CL were compared by 2D-PAGE in the pH range 4–7. From a total of 600 matched spots, 167 significantly altered protein spots in the CL, as compared to the POFs, were identified (average ratio >1.8, *P* < 0.05). Representative 2D gel images depicting significantly altered protein spots in the CL as compared to POF are shown in Figure 2 and the corresponding identified proteins along with the positive or negative fold change are listed in Supplementary Tables 3, 4, respectively. The identified proteins found upregulated in the CL as compared to POF included albumin (ALB), aldose reductase (AKR1B1), aldo-keto reductase family 1 member C4 (AKR1C4), fibrinogen gamma chain (FGG), isopentenyl-diphosphate delta isomerase 1 (IDI1), and fetuin (AHSG). Representative MS/MS spectra of some of the proteins that were differentially regulated between POFs and the CL in this study are shown in Figure 3 and Supplementary Figure 2. Functional annotation of differentially abundant proteins resulted in their association with top molecular and cellular functions including the inhibition of cell death, lipid metabolism, and cell migration (Figure 4A and Table 2). Furthermore, differentially abundant proteins in the CL were linked to canonical pathways that support the luteinization process and included geranylgeranyl diphosphate biosynthesis I (via Mevalonate), cholesterol biosynthesis, and RhoA signaling (Supplementary Figure 3 and Table 2). The IPA analysis of the significantly altered proteins further identified large number of upstream regulators that could be involved in luteinization-induced altered protein profiles in the CL. Top upstream regulators identified with a positive *z*-score (*z*-score > 2.0) included insulin growth factor-1 (IGF-1), nuclear factor erythroid derived (NFE2L2), sterol regulatory element-binding transcription factor 1 (SREBF1), and CCAAT/enhancer binding protein beta (CEBPB) (Figure 4B and Supplementary Table 5).

**Table 2:** IPA analysis overview: canonical pathways and molecular and cellular functions associated with differentially regulated proteins between POFs and the CL.

**Top molecular and cellular functions**	***P*-value**	**No. of molecules**	**Canonical pathways**	***P*-value**	**No. of molecules**
Cell death and survival	1.46E-10	45	Acute phase response signaling	8.39E-07	9
Steroid metabolism	6.21E-09	14	Super pathway of geranylgeranyldiphosphate biosynthesis (via mevalonate)	2.19E-06	4
Cellular movement	6.54E-07	39	Superpathway of cholesterol biosynthesis	1.80E-05	4
Molecular transport	7.09E-06	27	RhoA signaling	6.56E-05	6
Free radical scavenging	2.52E-05	13	LXR/RXR signaling	7.19E-05	6

### Western-Blot Analysis

**Figure 5:**
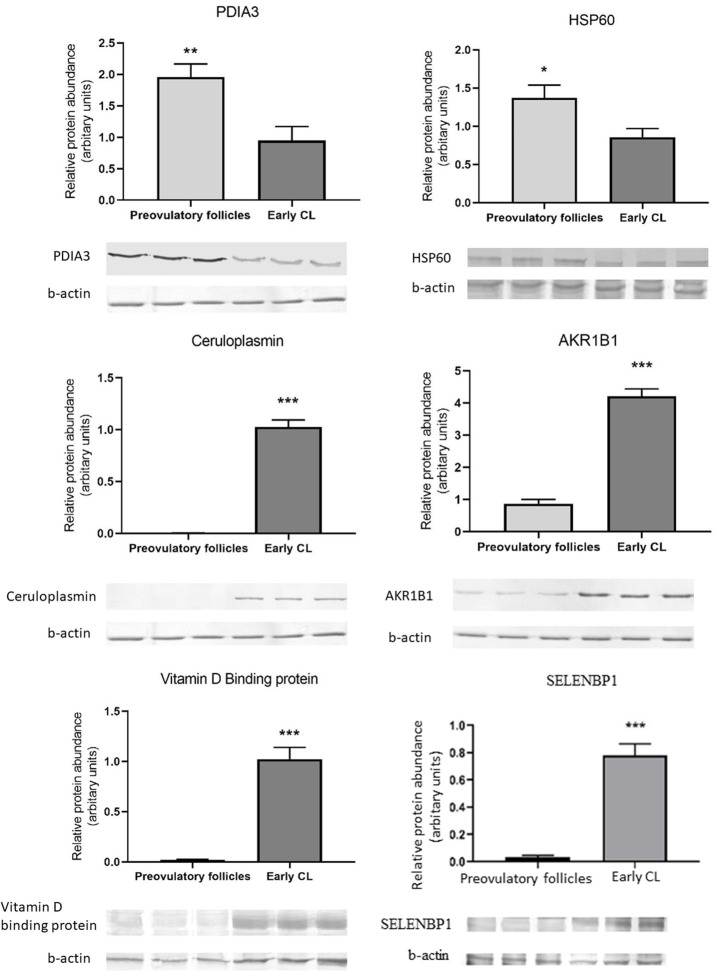
Western blot validation of change in abundances of proteins associated with unfolded protein resonse-PDIA3 and HSP60; oxidative stress response-Ceruloplasmin and AKR1B1; cell proliferation and migration-SELENBP1 and sterol transport-VDBP in newly formed CL as compared to preovulatory follicles. Data are presented as mean ± standard error. *P ≤ 0.05, **P ≤ 0.01, ***P ≤ 0.001 by *t*-test.

The 2D-PAGE results were further validated by Western-blot analysis. Six identified proteins that were either upregulated or downregulated in the CL or identified for the first time in the porcine CL (CP and VDBP) were selected for further analysis. Statistical analysis of protein levels relative to beta-actin (ACTB) showed similarities to 2D results. Whereas, an abundance of protein disulfide isomerase 3 and heat shock protein 60 were significantly decreased in CL, AKR1B1 was more abundant in the CL, and proteins CP, SELENBP1, and VDBP were detected only in the CL (Figure 5).

## Discussion

The aim of the present study was to determine the protein profile of POFs and evaluate the LH surge-induced changes in follicles leading to ovulation and CL formation. This is the first comprehensive proteomic characterization of the porcine POFs to the best of our knowledge that gives an insight into the functional processes associated with follicles. The follicular proteome was highly represented by cytoskeleton-associated proteins, the endoplasmic stress response, and the folding of proteins.

### Cytoskeletal Proteins

Cytoskeletal proteins were highly represented in POFs and included actin, tubulin, tropomyosin, and myosin. The genes corresponding to these proteins were previously described with luteinization of POFs (19). Cytoskeletal proteins play essential roles in changes associated with the differentiation of follicular cells. The expression of cytoskeletal proteins is under the control of FSH in rat granulosa cells, however, there are many reports highlighting the LH-induced morphological changes that involve the remodeling of the actin cytoskeleton, causing granulosa cells to become more rounded during the luteinization process and, more importantly, stimulation of P4 production (30–33). The other POF-associated structural proteins included microtubule-associated protein RP/EB1 (MAPRE1, spot 303 Supplementary Table 1) and vimentin. Vimentin existed in many isoforms (49–54 kDa) with a significantly decreased abundance in the CL. The expression of MAPRE1 did not show any significant difference between the POFs and the CL. In rat granulosa cells, an isoform of microtubule-associated proteins, MAP2D is expressed as an FSH responsive, and its expression is known to persist in the luteal cells (34). The MAP2D isoform binds to vimentin during follicular luteal transition. In mice, hCG induces phosphorylation of vimentin and its increased binding to low molecular weight MAP2D leading to vimentin disruption and contraction of granulosa cells which in turn results in an increase in the P4 synthesis (31). We speculate that, in pigs, the follicular expression of vimentin and MAPRE1, which binds to intermediate filaments, might have a similar function due to its reported involvement in follicle development (35).

### Endoplasmic Stress Response and Folding of Proteins

The other class of proteins with a high representation in POF was associated with the abrogation of endoplasmic reticulum (ER) stress. The development of a follicle to preovulatory stage is accompanied with an extensive proliferation of the granulosa cell, which leads to hypoxia (36). Such a condition may contribute to ER stress resulting in prosurvival unfolded protein response (UPR) signaling to maintain ER homeostasis (37). In the absence of UPR, ER stress can inhibit cell growth and result in apoptosis as a survival mechanism affecting P4 synthesis (38). The porcine POFs are under ER stress, as we observed POF-specific expression of 78 kDA glucose-regulated protein (GRP78), a central regulator of unfolded protein responses and an important marker of ER stress (39). Calreticulin and calumenin are calcium-binding chaperone proteins and their expression is induced as a result of UPR. Both these proteins were highly abundant in the POFs and are known to alleviate ER stress (40, 41). Additionally, calreticulin knock out female mice were shown to be infertile due to impaired folliculogenesis and decreased ovulatory rates (41), suggesting its importance in female reproduction.

The other class of proteins belonging to UPR that was expressed in POFs was heat shock proteins (HSPs). HSPs belong to the class of multifunctional chaperones that are expressed as a response to stress to protect cells and restore homeostasis (42). HSPs can prevent the incorrect assembly or folding of proteins, reduce apoptosis and increase cell proliferation and survival. We observed that abundances of HSP90, HSP70, and HSP60 were significantly higher in the POFs as compared to the CL, and these proteins existed in many isoforms in POFs. HSPs are known to alleviate the ER stress. In bovine granulosa cells, elevated temperatures induced ER stress, and, as a survival mechanism, an increase in GRP78, HSP70, and HSP90 was observed (43). GRP78 is an important regulator of steroidogenesis; it results in StAR activation at the mitochondria-associated ER membranes (44). The HSP90 also plays a regulatory role in oocyte maturation (45) and reduces stress-induced cell apoptosis by attenuating the caspase pathway (46). Therefore, the higher abundances of HSPs in POFs may be associated with their ability to survive the ER stress and guide the follicle toward ovulation. Interestingly, the expression of HSPs was significantly reduced in the newly formed CL. Heat shock resulting in elevated expression of HSP70 and HSP90 interferes with the steroid synthesis. It was shown that the expression of HSP70 is related to the downregulation of steroidogenic acute regulatory protein (StAR) and effects steroidogenesis in the rat luteal cells (47). These reports together with the results from present study suggest that whereas, HSPs have a protective role in POFs, there downregulation during luteinization is essential for P4 synthesis.

The LH surge causes differentiation of estrogen-producing follicular cells to the progesterone-producing luteal cells; a change in the abundance of proteins is therefore important for luteal function and survival. The proteins that were highly represented in newly formed CL were associated with biological functions that may be regulated by the LH surge.

### Steroid Biosynthesis and Metabolism

In this study, we observed that proteins associated with functional categories such as cholesterol biosynthesis/transport and steroid metabolism were differentially regulated between POFs and the newly formed CL. The cholesterol biosynthesis and metabolism was associated with an increase in the levels of StAR protein, IDI1, AKR1C4, and a decrease in estradiol 17-beta-dehydrogenase 1 (HSD17B1). The other proteins associated with cholesterol biosynthesis in porcine CL were FDPS and GGPS1. The importance of StAR in mediating the steroidogenic response is well documented (7, 48). The new finding of this study related to steroid biosynthesis was the activation of the mevalonate pathway through expression of HMGCS1, FDPS, GGPS1, and IDI1 (Supplementary Figure 4). HMGCS1 is a key rate-limiting enzyme in the cholesterol synthesis pathway, and its expression was recently reported in the bovine CL where it was suggested to support the production of high progesterone levels (49). The proteins of this pathway contain sterol response elements and, indeed, transcription factor SREBF1 was identified as an upstream regulator of proteins involved in steroid biosynthesis in our study. In rat ovaries, genes of the mevalonate pathway containing sterol response elements (SRE) were shown to be regulated by LH. It was suggested that LH-induced elevation in steroidogenesis results in decreased levels of cholesterol, which, in turn, activates the transcriptional induction of genes associated with cholesterol biosynthesis (50). IGF-1 regulated many of the proteins associated with steroid biosynthesis. Though the expression and role of IGF-1 has been extensively investigated in follicular growth and development, its role in luteal formation or development has not been investigated at length. However, IGF-I is reported to have steroidogenic actions in early pig corpora lutea (days 4–7 of the estrous cycle), which is lost on later days of the estrous cycle (51). The results of the present study suggest that LH induces cholesterol synthesis through the mevalonate pathway in the porcine CL to support progesterone production by the newly formed CL.

We also observed an increase in proteins involved in the sterol transport that included significantly higher expression of APOE, ALB, PITP, and VDBP (GC) in the CL as compared to POFs. Though the CL can produce cholesterol *de novo* as evidenced by the expression of HMGCS1, other mechanisms for obtaining cholesterol are required to meet the need for high progesterone production. This could be achieved by endocytosis of cholesterol-rich low-density lipoproteins (LDL) (52). VDBP belongs to the albumin superfamily, its expression was ~4.0-fold higher in the CL as compared to POFs, and this study reports its expression for the first time in the CL. IPA analysis grouped it with the proteins associated with cholesterol transport. The known functions of VDBP include its vitamin transport and actin remodeling, however, VDBP has also been shown to bind LDL (53), and we suggest that along with APOE it might transport LDL to the CL for luteal steroidogenesis.

### Oxidative Stress Response

The newly formed CL is under oxidative stress due to inflammation-like responses during ovulation after an LH surge and high steroidogenic activity. Inflammation results in the acute phase response (APR), and this was one of the most significant pathways associated with CL formation. We identified APR protein fetuin (AHSG), APR proteins with antioxidant functions, ceruloplasmin (CP), and haptoglobin (HP) proteoforms in the CL. The role of these proteins in the CL is not known, but AHSG was recently suggested to be a marker of luteinization (54). Oxidative stress is detrimental to the cells; it induces apoptosis, interferes with cholesterol transport, and is associated with luteal regression (55). We observed that proteins involved in the metabolism and scavenging of reactive oxygen species were upregulated in the CL as compared to POFs. The role of antioxidants in the control of CL function has been shown in many studies [reviewed in (55)]. Many of the antioxidant enzymes PRDX6, GSS, GPX3, and GLRX identified here play major roles in removing reactive oxygen species and were identified previously in porcine luteinized follicles (19). The other proteins belonging to anti-oxidative stress response included AKR1B1, ALDH2, CP, and GLO1. Aldo-keto reductases (AKR1B1) and GLO1 are induced in response to oxidative stress and detoxify carbonyl compounds resulting from lipid oxidation (56, 57). The detoxification of carbonyls might result from the binding between glutathione and aldo-keto reductases, resulting in alleviation of oxidative stress in female ovary (56). GLO1 is expressed ubiquitously in cells, and, recently, it has been reported to be expressed in mouse ovary and oocytes where it was shown to abrogate the glycative stress response through components of the SIRT1 functional network (58). Transcription factor NFE2L2 (Nrf2) was detected as the upstream regulator of many of the proteins involved in alleviating oxidative stress response. Nrf2 is an important cytoprotective factor that regulates critical antioxidant and stress-responsive genes. It was shown to be upregulated as a survival mechanism in bovine granulosa cells subjected to oxidative stress (59). Its role in the porcine luteal function is worth further investigation. These findings suggest that in the newly formed CL, besides the expression of well-known components of the antioxidation system, other antioxidants are also expressed to support survival of luteal cells and support P4 production.

### Cell Migration and Cell Survival

The other functional categories that were overrepresented in the CL were cell migration and cell survival. The luteinization and ovulation leading to the CL formation is associated with tissue remodeling, differentiation of follicular cells into large and small luteal cells, endothelial cell migration, and an intermixing of these cell types. These processes include modification of the extracellular matrix (ECM) and actin cytoskeleton. ECM remodeling is a interplay between proteinases and protease inhibitors. The expression of serine protease, plasminogen activator (PA), and the protease inhibitor system has been defined in pigs (60). Though uPA and the members of MMP and TIMP family are associated with the remodeling and angiogenesis of the CL in mice, the knock out of uPA did not result in significant changes in ovulation or CL function. It was suggested that other elements might be involved in remodeling during CL formation (7, 61). We observed that functional category cell migration was mainly associated with proteases CTSD and CTSH and protease inhibitors SERPINA3-6 and SERPINA3-8, which were more abundant in the CL. Some of the isoforms of SERPINs were detected only in the CL. Cystein cathepsins and serine proteases are known to degrade extarcellular cellular matrix facilitating ovulation, cell migration, and angiogenesis during CL formation (7, 62). The newly formed CL undergoes extensive tissue growth and vascularization and a balance between proteases and protease inhibitors is important to regulate the proteolysis that could otherwise damage tissue growth and it vascularization. A CL-specific expression of SELENBP1 was observed in this study. The functions of SELENBP1 are largely unknown; however, it is widely investigated in tumor tissues where it is expressed at lower levels, including in ovarian cancer models (63). It is reported to play a role in tumor suppression by controlling cellular proliferation and migration (64). CL formation also involves the fine-tuning of cell proliferation and cell survival after ovulation. It was observed that, whereas proteins promoting cell death, such as PDCD6 and DIABLO, were downregulated, proteins promoting luteal survival shown to be associated with proteasome subunits were upregulated in the CL. Proteasomes have complex functions, the proteasome-ubiquitin system is also a regulator of proteolytic enzymes, such as MMPs. The inhibitors of proteasomes result in apoptosis in proliferating cells and, on the other hand, in dying cells the same inhibitors can block apoptosis (65). This observation explains their role in both CL development and CL regression.

**Figure 6:**
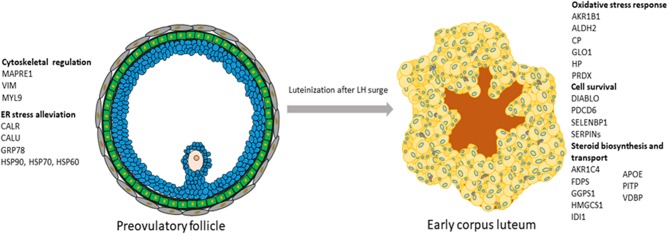
Schematic representation of the processes and their associated proteins that were highly represented either in preovulatory follicles or in early corpus luteum.

In conclusion, a number of proteins characterizing POFs were identified. It can be suggested that, in POFs, cytoskeletal proteins, such as vimentin and MAPRE1, are not only associated with changing the shape of follicular cells but also with steroidogenesis. A number of proteins and biological pathways associated with the differentiation of POFs to CL were identified. Some of the proteins that might be regulated by LH and associated with steroidogenesis in the newly formed porcine CL, such as IDI1, FDPS, and HMGCS1, and cell survival, such as the eukaryotic translation factor, PDCD6, DIABLO, and the proteasome ubiquitin system, are reported in this study, thereby providing novel insights into peri-ovulatory regulation of proteins that might play a role in luteinization processes and luteal survival (Figure 6). As in bovines (54), the induction of AHSG with the formation of CL observed here can be suggested to be marker of luteinization.

## Data Availability Statement

All datasets generated for this study are included in the article/Supplementary Material.

## Ethics Statement

All procedures involving the use of animals were approved by the Animal Ethics Committee, University of Warmia and Mazury in Olsztyn, Poland, and were conducted in accordance with the national guidelines for agricultural animal care.

## Author Contributions

BM designed the experiments. PL and BM performed the experiments. BM, PL, and DS analyzed the results. BM and DS wrote the manuscript. All authors reviewed the manuscript.

### Conflict of Interest

The authors declare that the research was conducted in the absence of any commercial or financial relationships that could be construed as a potential conflict of interest.
