# Supplementary material for: Proteomic Analysis of Porcine Pre-ovulatory Follicle Differentiation Into Corpus Luteum
Source: Front Endocrinol (Lausanne). 2019 Nov 15;10:774. doi: 10.3389/fendo.2019.00774 (PMC6879000; doi:10.3389/fendo.2019.00774)
Supplement: Supplementary file 3 [file Table_3.DOCX]

Supplementary Table S3. Proteins whose abundace increased significantly in CL as compared to POFs

| Spot no. | Identified protein | p-Value | Fold change | NCBI accession number | MW (kDa/PI) |
| --- | --- | --- | --- | --- | --- |
| 7 | protein-glutamine gamma-glutamyltransferase 2 | 0,0094 | 1,98 | 545880563 | 79/5.23 |
| 11 | NADH-ubiquinone oxidoreductase 75 kDa subunit | 0,0053 | 2,66 | 311272935 | 75/5.79 |
| 12 | Ceruloplasmin precursor | 0,0031 | 2,63 | 406647880 | 122/ |
| 29 | albumin | 0,0001 | 3,9 | 51235682 | 71/5.92 |
| 30 | albumin | 0,0001 | 3,91 | 51235682 | 71/5.92 |
| 31 | albumin | 0,0015 | 7,76 | 51235682 | 71/5.92 |
| 32 | albumin | 0,0001 | 9,58 | 51235682 | 71/5.92 |
| 33 | albumin | 0,0005 | 6,07 | 51235682 | 71/5.92 |
| 34 | albumin | 0,0011 | 8,78 | 51235682 | 71/5.92 |
| 35 | hyaluronidase | 0,0001 | 9,34 | 1708183 | 52/6.59 |
| 36 | albumin | 0,0001 | 9,59 | 51235682 | 71/5.92 |
| 37 | serpin A3-6 | 0,0001 | 4,86 | 927163661 | 47/5.75 |
| 38 | serpin A3-6 | 0,0001 | 3,52 | 927163661 | 47/5.75 |
| 39 | serpin A3-6 | 0,0001 | 3,03 | 927163661 | 47/5.8 |
| 40 | serpin A3-6 | 0,0002 | 3,92 | 927163661 | 47/5.75 |
| 41 | serpin A3-8 | 0,0002 | 3,61 | 350587171 | 49/6.09 |
| 42 | serpin A3-8 | 0,0016 | 4,88 | 350587171 | 49/6.09 |
| 45 | alpha-2-HS-glycoprotein | 0,0072 | 1,96 | 545865183 | 40/5.5 |
| 46 | alpha-2-HS-glycoprotein | 0,0042 | 3,22 | 545865183 | 40/5.5 |
| 47 | alpha-2-HS-glycoprotein | 0,0001 | 4,87 | 545865183 | 40/5.5 |
| 48 | alpha-2-HS-glycoprotein | 0,0001 | 4,14 | 545865183 | 40/5.5 |
| 49 | prolyl 4-hydroxylase beta polypeptide | 0,0001 | 5,61 | 358009193 | 56/4.78 |
| 52 | serpin A3-8 | 0,0001 | 4,45 | 927163614 | 47/5.8 |
| 53 | serpin A3-6 | 0,0001 | 4,69 | 194038353 | 47/5.8 |
| 54 | serpin A3-8 | 0,0001 | 3,69 | 927163614 | 47/5.8 |
| 55 | serpin A3-6 | 0,0121 | 2,01 | 194038353 | 47/5.8 |
| 57 | serpin A3-6 | 0,0001 | 3,19 | 194038353 | 47/5.8 |
| 58 | vitamin D-binding protein | 0,0003 | 4,27 | 255068732 | 55/5.38 |
| 60 | antithrombin-III precursor | 0,0015 | 2,8 | 194018664 | 53/5.84 |
| 66 | 3-hydroxy-3-methylglutaryl-CoA synthase 1 | 0,0016 | 1,83 | 356582301 | 58/5.37 |
| 73 | glutathione synthetase | 0,0001 | 2,21 | 347543782 | 54/5.74 |
| 109 | eukaryotic initiation factor 4A-I | 0,0028 | 2,16 | 154147660 | 46/5.32 |
| 118 | fibrinogen gamma chain precursor | 0,0088 | 3,01 | 347300308 | 50/5.98 |
| 119 | fibrinogen gamma chain precursor | 0,0014 | 2,28 | 347300308 | 50/5.98 |
| 122 | fibrinogen gamma chain precursor | 0,0037 | 2,24 | 347300308 | 50/5.98 |
| 123 | aldehyde dehydrogenase, mitochondrial precursor | 0,0058 | 1,97 | 113205888 | 57/6.43 |
| 131 | cytosol aminopeptidase-like | 0,0014 | 1,97 | 350587377 | 25/6.54 |
| 135 | selenium binding protein 1 | 0,0021 | 6,1 | 927122403 | 53/6.17 |
| 147 | ribonuclease inhibitor | 0,013 | 2,25 | 132574 | 51/4.76 |
| 161 | gamma-enolase isoform 1 | 0,0001 | 2,6 | 335288522 | 47/4.9 |
| 172 | ornithine decarboxylase-like | 0,0015 | 1,91 | 350585823 | 51/4.9 |
| 173 | haptoglobin | 0,0005 | 2,33 | 189409353 | 39/6.51 |
| 174 | haptoglobin | 0,0002 | 5,04 | 189409353 | 39/6.51 |
| 184 | haptoglobin precursor | 0,0314 | 2,77 | 47522826 | 39/6.51 |
| 185 | farnesyl pyrophosphate synthase precursor | 0,0007 | 1,95 | 297591959 | 49/6.06 |
| 193 | nuclear distribution gene C homolog | 0,0074 | 1,93 | 345091002 | 38/5.34 |
| 194 | nuclear distribution gene C homolog | 0,0026 | 2,1 | 345091002 | 38/5.34 |
| 216 | tropomyosin beta chain | 0,0001 | 5,41 | 194018702 | 33/4.62 |
| 217 | tropomyosin alpha-1 chain isoform X3 | 0,0175 | 2,12 | 545800038 | 37/4.67 |
| 218 | annexin I | 0,0145 | 2,09 | 1165145 | 38/6.47 |
| 220 | clusterin | 0,0005 | 1,8 | 3023523 | 52/5.62 |
| 225 | V-type proton ATPase subunit d 1 | 0,0025 | 2,16 | 311257142 | 41/4.89 |
| 228 | clusterin precursor | 0,11 | 1,83 | 47522770 | 52/5.62 |
| 229 | catechol O-methyltransferase | 0,0258 | 1,85 | 305855180 | 30/5.38 |
| 232 | farnesyl diphosphate synthase | 0,0007 | 3,23 | 262072800 | 41/5.2 |
| 233 | eukaryotic translation initiation factor 2 subunit 1 | 0,0012 | 1,99 | 194038445 | 36/5.23 |
| 235 | farnesyl pyrophosphate synthase precursor | 0,0007 | 5,08 | 297591959 | 49/6.06 |
| 239 | aldo-keto reductase family 1 member C4 | 0,0021 | 5,6 | 178056496 | 37/7.62 |
| 241 | glyceraldehyde-3-phosphate dehydrogenase | 0,0012 | 2,87 | 112980811 | 28/6.77 |
| 243 | suppressor of G2 allele of SKP1 homolog | 0,006 | 1,84 | 545855060 | 38/5.39 |
| 246 | serine/threonine-protein phosphatase 2A catalytic subunit alpha | 0,024 | 1,91 | 47523790 | 36/5.3 |
| 247 | serine/threonine-protein phosphatase 2A catalytic subunit beta | 0,0009 | 2,34 | 1352666 | 34/5.45 |
| 249 | serine/threonine-protein phosphatase 2A catalytic subunit beta | 0,0017 | 2,89 | 1352666 | 34/5.45 |
| 251 | immunoglobulin-binding protein 1 isoform 2 | 0,0046 | 1,81 | 194045136 | 39/5.21 |
| 255 | glutaredoxin 3 | 0,0001 | 1,98 | 345199274 | 37/5.65 |
| 256 | glutaredoxin 3 | 0,0017 | 5,03 | 345199274 | 37/5.65 |
| 257 | F-actin capping protein subunit alpha 1 | 0,0025 | 1,88 | 147899312 | 33/5.53 |
| 262 | transaldolase | 0,0151 | 2,88 | 349732238 | 37/6.28 |
| 271 | macrophage-capping protein-like isoform 3 | 0,0024 | 3,86 | 311252249 | 39/5.88 |
| 275 | aldose reductase | 0,0081 | 4,06 | 48374071 | 36/5.89 |
| 278 | actin-related protein 2 | 0,0003 | 3,01 | 197251934 | 45/6.3 |
| 279 | leukocyte elastase inhibitor | 0,001 | 1,94 | 417185 | 43/5.98 |
| 280 | trans-1,2-dihydrobenzene-1,2-diol dehydrogenase | 0,0027 | 2,62 | 47523420 | 30/6.34 |
| 281 | aldose 1-epimerase | 0,0003 | 2,25 | 47523866 | 38/6.31 |
| 283 | apolipoprotein E precursor | 0,0368 | 2,45 | 47523674 | 37/5,62 |
| 288 | enolase-phosphatase E1 | 0,0348 | 1,95 | 345091086 | 29/4.74 |
| 292 | elongation factor 1-delta-like isoform 2 | 0,0149 | 4,5 | 545837896 | 29/4.86 |
| 295 | EF-hand domain-containing protein D2-like | 0,0315 | 1,91 | 311258550 | 26/5.15 |
| 300 | tubulin-folding cofactor B-like | 0,0019 | 2,08 | 329663924 | 28/5.23 |
| 302 | spermidine synthase | 0,0066 | 1,94 | 350585594 | 35/5.33 |
| 306 | phenol sulfotransferase | 0,0011 | 1,8 | 47523314 | 34/5.72 |
| 311 | elongation factor 1-beta | 0,0016 | 1,86 | 343488474 | 25/4.51 |
| 313 | tropomyosin 4 | 0,0001 | 2,02 | 3661527 | 28/4.67 |
| 317 | EF-hand domain-containing protein D2 | 0,0461 | 2,38 | 311258550 | 27/5.15 |
| 320 | proteasome subunit alpha type-3 isoform 2 | 0,0029 | 1,83 | 194034201 | 27/5.29 |
| 322 | chloride intracellular channel protein 5 isoform X2 | 0,008 | 1,87 | 545839994 | 29/5.54 |
| 335 | cathepsin D protein | 0,0039 | 1,9 | 56417363 | 43/7.67 |
| 340 | phosphatidylinositol transfer protein | 0,0001 | 3,2 | 545859572 | 32/6.12 |
| 341 | eukaryotic translation initiation factor | 0,0007 | 3,67 | 48675941 | 27/4.56 |
| 351 | ubiquitin carboxyl-terminal hydrolase isozyme L3 | 0,008 | 1,97 | 116175277 | 26/4.83 |
| 356 | rho GDP-dissociation inhibitor 1 | 0,0011 | 2,11 | 319401915 | 23/5.12 |
| 357 | NAD(P)H-hydrate epimerase | 0,0347 | 1,8 | 122133333 | 31/7.57 |
| 367 | oligoribonuclease, mitochondrial-like | 0,0001 | 2,35 | 311263942 | 27/7.01 |
| 369 | putative ATP-dependent Clp protease proteolytic subunit, mitochondrial-like | 0,0046 | 1,97 | 350580642 | 22/5.59 |
| 372 | oligoribonuclease, mitochondrial-like | 0,0048 | 1,83 | 311263942 | 27/7.01 |
| 374 | cathepsin H transcript variant 3 | 0,0021 | 1,86 | 172050735 | 28/6.21 |
| 378 | peroxiredoxin-6 | 0,0074 | 2,77 | 47523870 | 25/5.73 |
| 379 | steroidogenic acute regulatory protein, mitochondrial isoform X1 | 0,0001 | 2,81 | 56711334 | 32/9.07 |
| 380 | pyridoxine-5'-phosphate oxidase | 0,0007 | 1,8 | 927196214 | 30/8.49 |
| 381 | isopentenyl-diphosphate Delta-isomerase 1 | 0,0005 | 4,69 | 545854135 | 33/8.1 |
| 382 | inorganic pyrophosphatase 2, mitochondrial-like | 0,0015 | 1,9 | 311262725 | 24/7.01 |
| 383 | cathepsin D protein | 0,0002 | 2,2 | 56417363 | 43/7.67 |
| 384 | proteasome subunit alpha type-6 isoform 1 | 0,0005 | 2,9 | 6755198 | 28/6.34 |
| 385 | heat shock protein beta-1 | 0,0001 | 5,89 | 55926209 | 23/6.23 |
| 388 | translationally-controlled tumor protein | 0,0001 | 2,23 | 47523802 | 19/4.84 |
| 393 | lactoylglutathione lyase isoform 1 | 0,0001 | 2,27 | 194040450 | 21/5.22 |
| 394 | peroxiredoxin-2 | 0,0001 | 1,92 | 347300176 | 22/5.23 |
| 407 | proteasome subunit beta type-3 | 0,0015 | 1,96 | 222136592 | 23/5.76 |
| 414 | ig kappa chain V-II region RPMI 6410 | 0,0051 | 1,98 | 559775234 | 27/8.23 |
| 415 | mitochondrial import receptor subunit TOM22 homolog | 0,0254 | 1,95 | 311255064 | 15/4.29 |
| 422 | acireductone dioxygenase 1 | 0,0022 | 1,82 | 343887450 | 21/5.07 |
| 428 | retinol-binding protein 4 | 0,0066 | 2,38 | 47522930 | 23/5.41 |
| 431 | ferritin L subunit | 0,0001 | 2,86 | 10304378 | 18/5.79 |
| 434 | glutathione peroxidase 3 precursor | 0,0003 | 4,2 | 169646366 | 26/8.78 |
| 441 | coactosin-like protein-like | 0,0004 | 2,9 | 335309813 | 17/5.23 |
