# Supplementary material for: Proteomic Analysis of Porcine Pre-ovulatory Follicle Differentiation Into Corpus Luteum
Source: Front Endocrinol (Lausanne). 2019 Nov 15;10:774. doi: 10.3389/fendo.2019.00774 (PMC6879000; doi:10.3389/fendo.2019.00774)
Supplement: Supplementary file 4 [file Table_4.DOCX]

Supplementary Table S4. Proteins whose abundace decreased significantly in CL as compared to POFs

| Spot no. | Identified protein | p-Value | Fold change | NCBI accession number | MW (kDa/PI) |
| --- | --- | --- | --- | --- | --- |
| 2 | 94 kDa glucose-regulated protein | 0,0004 | -2,7 | 33301108 | 93/4.75 |
| 4 | heat shock protein HSP 90-alpha | 0,0018 | -1,89 | 47522774 | 85/4.93 |
| 13 | neutral alpha-glucosidase AB precursor | 0,0345 | -1,92 | 47522680 | 108/5.64 |
| 22 | 78 kDa glucose-regulated protein | 0,0012 | -2,73 | 927099357 | 73/5.43 |
| 23 | 78 kDa glucose-regulated protein | 0,0005 | -2,87 | 927099357 | 73/5.43 |
| 62 | 60 kDa heat shock protein, mitochondrial | 0,0006 | -2,28 | 359811347 | 61/5.7 |
| 75 | T-complex protein 1 subunit theta-like isoform 3 | 0,0015 | -1,83 | 545881741 | 58/5.24 |
| 77 | protein disulfide-isomerase A3 precursor | 0,0033 | -1,91 | 304365428 | 57/5.93 |
| 85 | histone-binding protein RBBP4 | 0,0001 | -2,98 | 417515920 | 47/4.74 |
| 86 | protein kinase, cAMP-dependent, regulatory, type II, beta | 0,0005 | -2,75 | 1191907144 | 45/4.82 |
| 87 | cAMP-dependent protein kinase type II-alpha regulatory subunit | 0,0014 | -2,03 | 125195 | 45/4.82 |
| 88 | cAMP-dependent protein kinase type II-alpha regulatory subunit | 0,0041 | -1,82 | 125195 | 45/4.82 |
| 89 | cAMP-dependent protein kinase type II-alpha regulatory subunit | 0,0001 | -4,09 | 125195 | 45/4.82 |
| 101 | 26S proteasome non-ATPase regulatory subunit 5 | 0,0369 | -1,87 | 350579579 | 46/5.08 |
| 112 | TXNDC5 protein | 0,0022 | -2,05 | 927206299 | 46/5.32 |
| 113 | septin 2 | 0,0089 | -2,34 | 345090969 | 41/6.19 |
| 114 | ruvB-like 2 | 0,0136 | -2,19 | 345110630 | 51/5.49 |
| 129 | alpha-enolase | 0,0224 | -2,01 | 927145216 | 47/6.44 |
| 133 | guanosine diphosphate dissociation inhibitor 2 | 0,0325 | -1,97 | 45758488 | 51/6.31 |
| 137 | reticulocalbin 2, EF-hand calcium binding domain precursor | 0,0001 | -5,43 | 346644882 | 36/4.22 |
| 139 | calumenin | 0,0001 | -9,18 | 350595262 | 37/4.49 |
| 140 | calumenin | 0,0015 | -2,49 | 350595262 | 37/4.49 |
| 141 | calumenin | 0,0084 | -3,32 | 350595262 | 37/4.49 |
| 142 | calumenin | 0,0012 | -1,96 | 350595262 | 37/4.49 |
| 143 | calumenin | 0,0029 | -1,82 | 350595262 | 37/4.49 |
| 145 | reticulocalbin-3 | 0,0001 | -2,49 | 927141966 | 37/4.76 |
| 148 | vimentin | 0,0031 | -2,69 | 753702890 | 53/5.06 |
| 149 | vimentin | 0,0001 | -2,48 | 753702890 | 53/5.06 |
| 150 | vimentin | 0,0001 | -3,07 | 753702890 | 54/5.06 |
| 152 | vimentin | 0,0022 | -3,16 | 753702890 | 53/5.06 |
| 154 | vimentin | 0,0001 | -2,74 | 753702890 | 54/5.06 |
| 155 | vimentin | 0,0001 | -2,38 | 753702890 | 49/5.09 |
| 157 | vimentin | 0,0001 | -2,39 | 753702890 | 49/5.09 |
| 166 | heat shock cognate 71 kDa protein-like | 0,0481 | -1,85 | 350588578 | 59/5.16 |
| 205 | succinyl-CoA ligase [ADP-forming] subunit beta, mitochondrial | 0,0288 | -1,96 | 311266259 | 50/7.57 |
| 214 | protein SET | 0,0025 | -2,13 | 346644699 | 32/4.12 |
| 230 | estradiol 17-beta-dehydrogenase 1 | 0,0009 | -5,41 | 190360573 | 35/4.96 |
| 269 | isocitrate dehydrogenase [NAD] subunit alpha, mitochondrial | 0,0008 | -3,28 | 335292262 | 40/6.72 |
| 272 | 3-hydroxyisobutyryl-CoA hydrolase, mitochondrial-like | 0,0079 | -2,12 | 350593717 | 43/6.23 |
| 273 | NADH dehydrogenase [ubiquinone] 1 alpha subcomplex subunit 10, mitochondrial | 0,0036 | -1,81 | 311273371 | 41/6.77 |
| 309 | mitochondrial complement component 1 Q subcomponent-binding protein | 0,0045 | -1,98 | 770114232 | 31/4.67 |
| 326 | prohibitin | 0,0095 | -1,81 | 927196484 | 30/5.57 |
| 347 | vimentin-like | 0,0004 | -7,08 | 753702890 | 53/5.06 |
| 387 | chromobox protein homolog 1 | 0,0162 | -3,11 | 311267482 | 21/4.85 |
| 411 | heat shock protein 27 | 0,0001 | -2,39 | 55926209 | 23/6.23 |
| 417 | myosin regulatory light polypeptide 9 | 0,0056 | -2,8 | 347300174 | 20/4.8 |
| 418 | diablo homolog, mitochondrial-like | 0,0078 | -3,24 | 927204630 | 26/8.81 |
| 419 | programmed cell death protein 6 | 0,0058 | -1,82 | 927226295 | 22/5.16 |
| 421 | chromobox protein homolog 3-like isoform 2 | 0,0264 | -2,05 | 545883582 | 20/5.03 |
| 429 | ATP synthase, H+ transporting, mitochondrial Fo complex,  subunit d | 0,0006 | -2,02 | 347658971 | 18/5.99 |
| 435 | myosin light chain isoform LC17a | 0,0001 | -2,93 | 253577 | 17/4.56 |
| 446 | chloride intracellular channel protein 4-like | 0,0001 | -2,03 | 350585766 | 15/5.91 |
