# Supplementary material for: Proteomic Analysis of Porcine Pre-ovulatory Follicle Differentiation Into Corpus Luteum
Source: Front Endocrinol (Lausanne). 2019 Nov 15;10:774. doi: 10.3389/fendo.2019.00774 (PMC6879000; doi:10.3389/fendo.2019.00774)
Supplement: Supplementary file 6 [file Presentation_1.PPTX]

## Slide 1
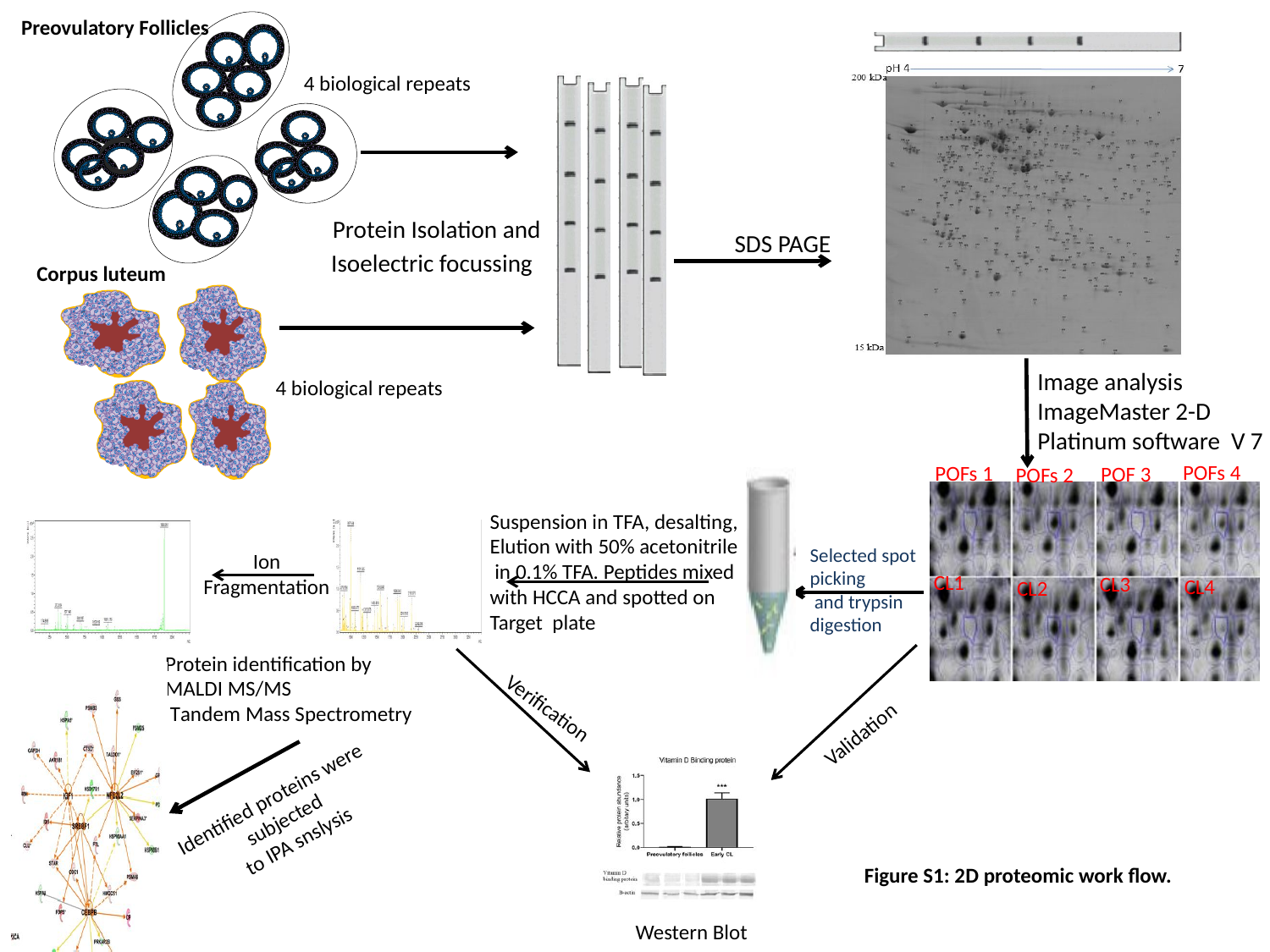

Preovulatory Follicles
4 biological repeats
Protein Isolation and
SDS PAGE
Isoelectric focussing
Corpus luteum
Image analysis
ImageMaster 2-D
Platinum software V 7
4 biological repeats
POFs 4
POFs 1
POF 3
POFs 2
CL1
CL3
CL4
CL2
Suspension in TFA, desalting,
Elution with 50% acetonitrile
 in 0.1% TFA. Peptides mixed
with HCCA and spotted on
Target plate
Selected spot picking
 and trypsin digestion
Ion
Fragmentation
Protein identification by MALDI MS/MS
 Tandem Mass Spectrometry
Verification
Validation
Identified proteins were
subjected
 to IPA snslysis
Figure S1: 2D proteomic work flow.
Western Blot

## Slide 2
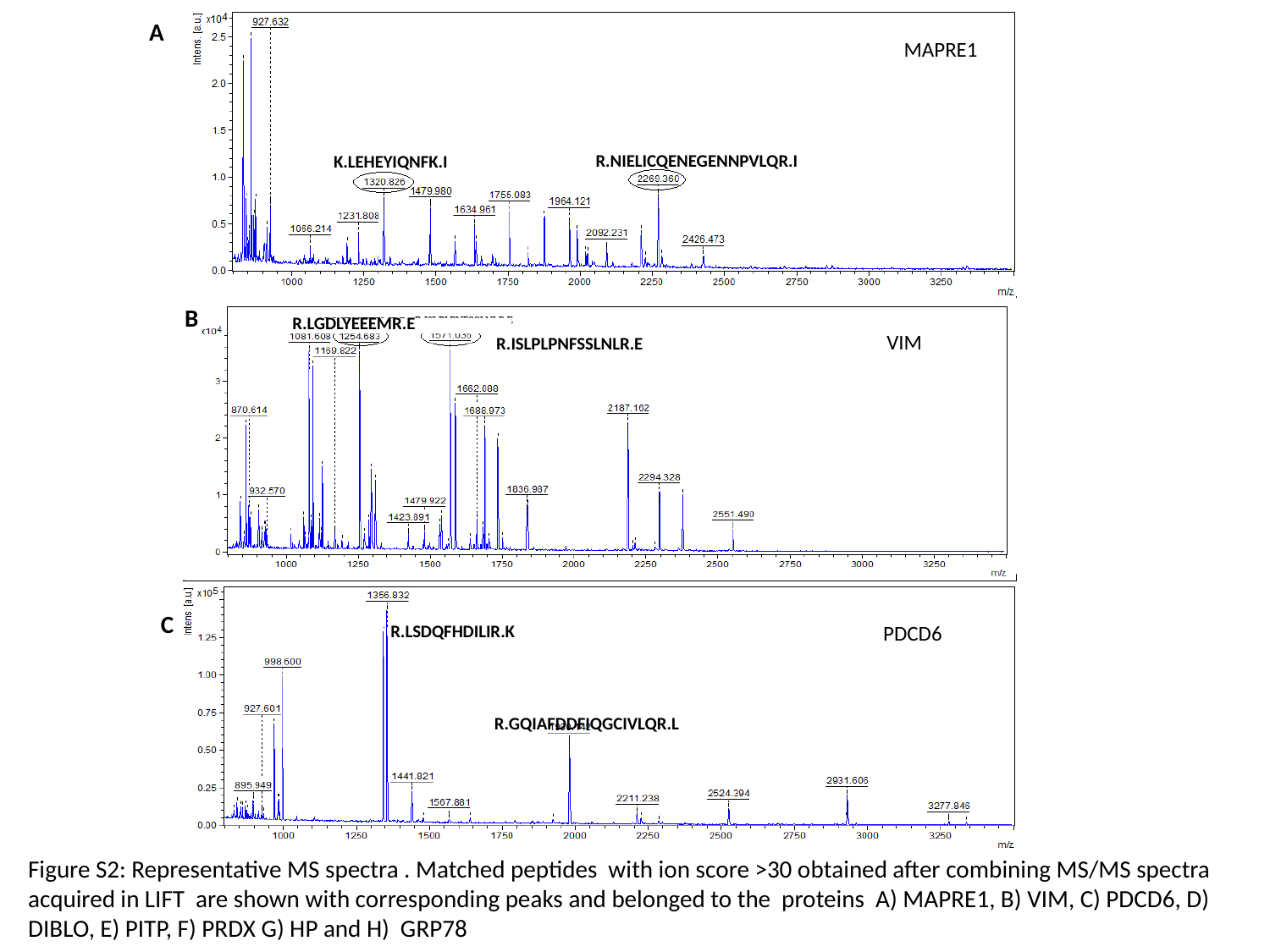

A
MAPRE1
R.NIELICQENEGENNPVLQR.I
K.LEHEYIQNFK.I
R.LGDLYEEEMR.E
VIM
R.ISLPLPNFSSLNLR.E
R.LSDQFHDILIR.K
R.GQIAFDDFIQGCIVLQR.L
PDCD6
B
C
K.AGVNFSEFTGVWK.Y
Figure S2: Representative MS spectra . Matched peptides with ion score >30 obtained after combining MS/MS spectra acquired in LIFT are shown with corresponding peaks and belonged to the proteins A) MAPRE1, B) VIM, C) PDCD6, D) DIBLO, E) PITP, F) PRDX G) HP and H) GRP78

## Slide 3
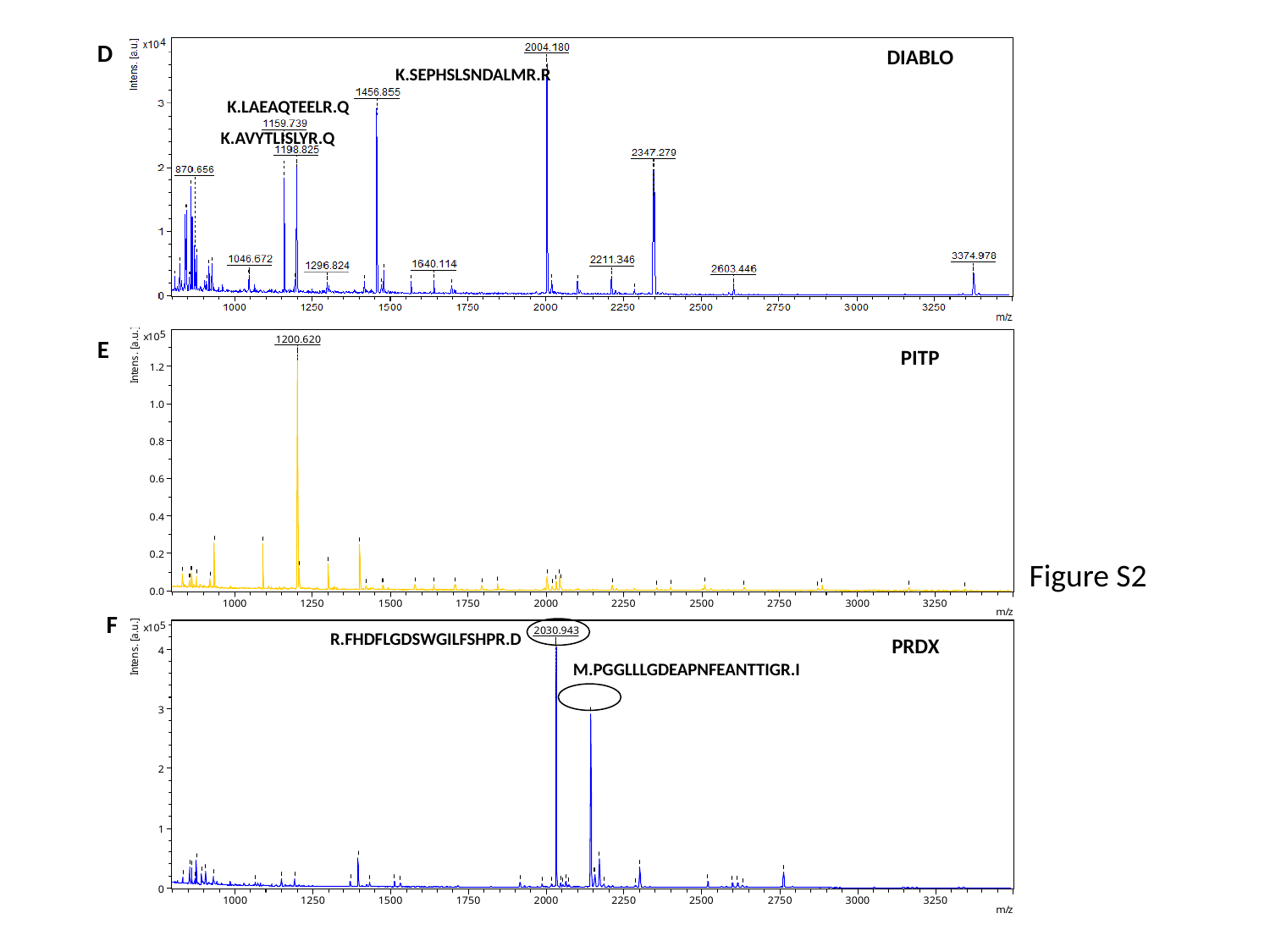

D
K.SEPHSLSNDALMR.R
K.LAEAQTEELR.Q
K.AVYTLISLYR.Q
DIABLO
PITP
R.FHDFLGDSWGILFSHPR.D
M.PGGLLLGDEAPNFEANTTIGR.I
PRDX
E
K.AWNAYPYCR.T
K.HVEAVYIDIADR.S
Figure S2
F

## Slide 4
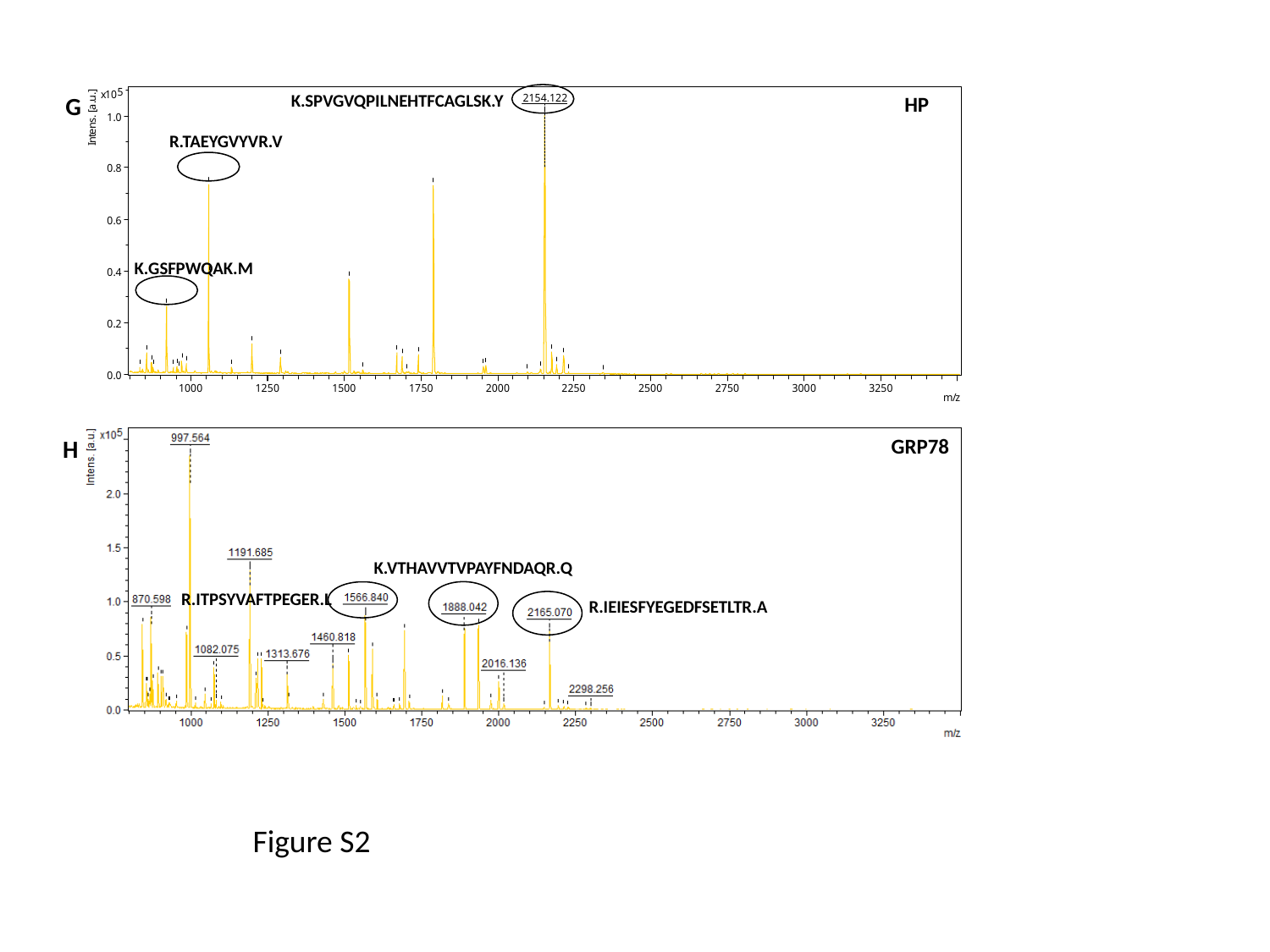

K.SPVGVQPILNEHTFCAGLSK.Y
R.TAEYGVYVR.V
K.GSFPWQAK.M
HP
G
GRP78
K.VTHAVVTVPAYFNDAQR.Q
R.ITPSYVAFTPEGER.L
R.IEIESFYEGEDFSETLTR.A
H
Figure S2

## Slide 5
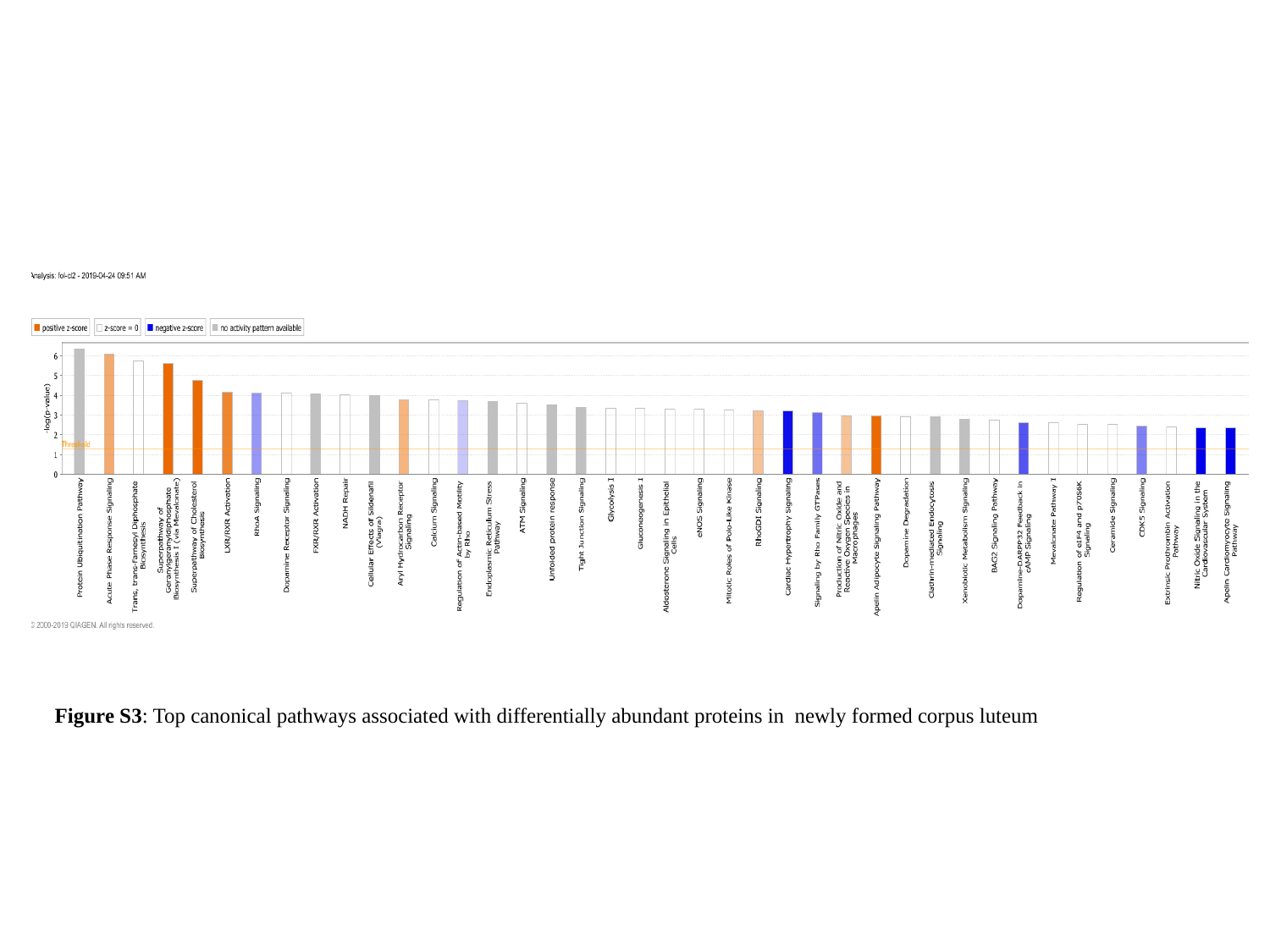

Figure S3: Top canonical pathways associated with differentially abundant proteins in newly formed corpus luteum

## Slide 6
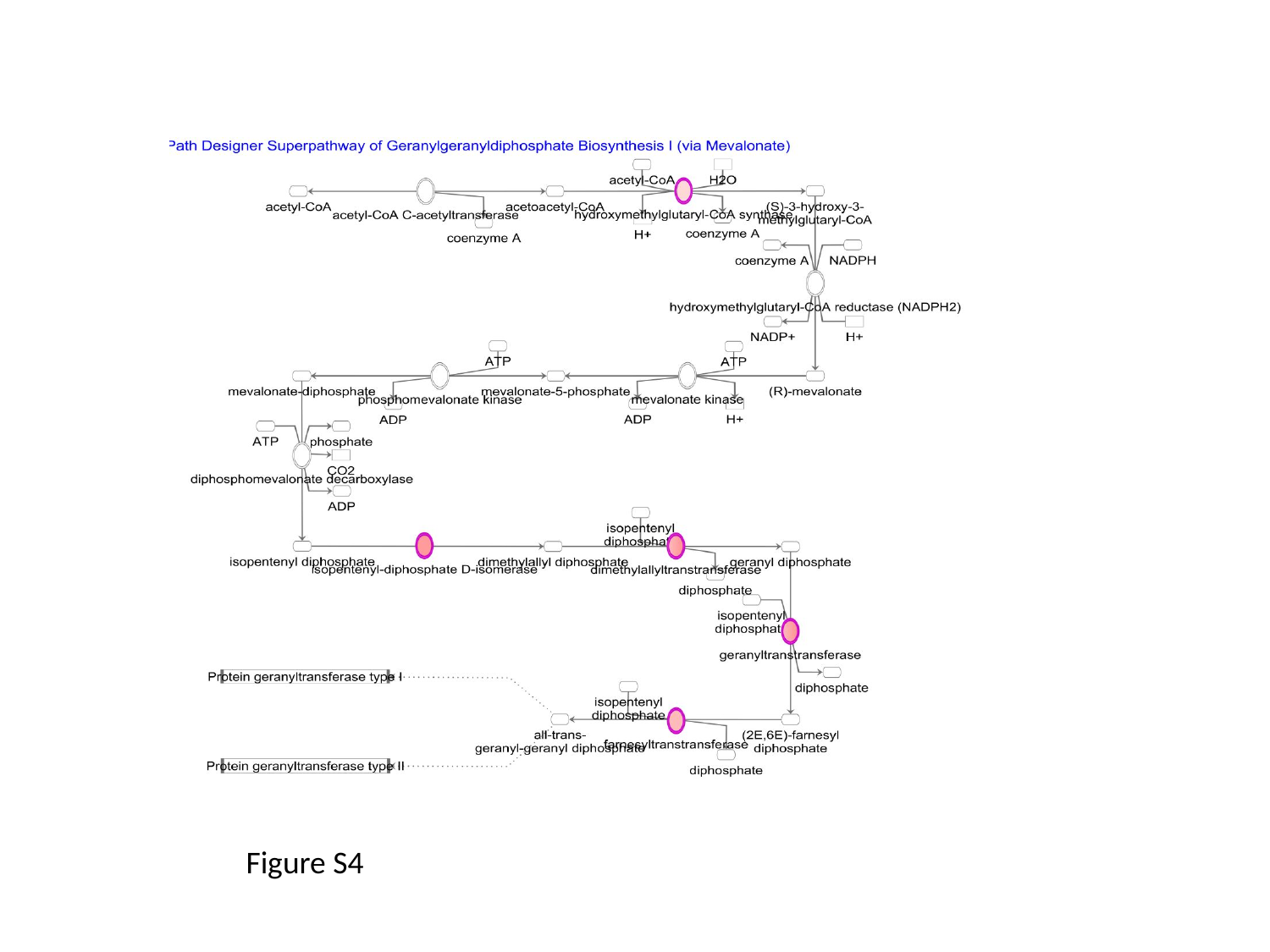

Figure S4
